# CCL20 in the tumor microenvironment: implications for cancer progression and therapeutic approaches

**DOI:** 10.1007/s12094-025-03874-5

**Published:** 2025-02-22

**Authors:** Louis Boafo Kwantwi, James Danquah Boafo, Bevelyn Emefa Egleh, Mingfeng Li

**Affiliations:** 1 Department of Biomedical Sciences, College of Medicine, Northeast Ohio Medical University, Rootstown, OH 44272 USA; 2 Department of Nursing and Midwifery, Faculty of Health and Allied Sciences, Pentecost University, Sowutoum, Ghana; 3 Department of Biomedical Sciences, College of Arts and Sciences, Kent State University, Kent, OH 44242 USA; 4 Department of Pathology, Affiliated Subei People’S Hospital of Yangzhou University, Yangzhou, 225000 Jiangsu China

**Keywords:** Immune cells, Tumor cells, Tumor microenvironment, CCL20, CCR6, Endothelial cells, Fibroblast

## Abstract

Increasing knowledge of the immunosuppressive tumor microenvironment in cancer-related processes has led to the developing of novel immune-based therapies that have changed the cancer treatment paradigm. In the tumor microenvironment, the plethora of soluble factors secreted by tumor cells interacts with immune cells and non-immune components to deliver signals necessary for tumor progression. Accordingly, targeting tumor-derived factors inducing this immunosuppressive tumor microenvironment has become an appealing therapeutic potential in advancing cancer treatment. CCL20, a chemokine best known to induce leucocyte migration in response to pathological and inflammatory conditions, has been implicated in tumor proliferation, angiogenesis, metastasis, immunosuppression, and therapeutic resistance. Notably, CCL20 and its receptor CCR6 are important in tumor microenvironment interactions. This review discusses the interaction between the CCL20–CCR6 axis and the tumor microenvironment and how these interactions promote tumor progression. Also, an outline of studies utilizing CCL20 in combination with other standard cancer treatments has been shed.

## Introduction

Chemokines are small secretory cytokines classified into C, CC, CXC, and CX3C based on the positioning of the conserved cysteine residue[1, 2]. Upon binding to their cognate receptors, chemokines direct immune tracking and play an important role in lymphoid tissue development[3–5]. Evidence has shown that dysregulation of chemokines triggers several inflammatory-mediated pathological conditions including cancers[6–8]. Chemokines stimulate tumor proliferation, metastasis, angiogenesis, and therapeutic resistance by promoting infiltration of immunosuppressive cells to tumor sites[9–12]. Besides, chemokines interact with non-immune cells including endothelial cells, tumor cells, and fibroblasts to promote tumor progression[13].

CCL20 is a member of the family of cysteine–cysteine and the subfamily of alpha chemokines. Although chemokines are known to be unselective in their binding to receptors, CCL20 is an exception as it binds solely to the CCR6 receptor [14, 15]. Although CCL20 exerts antitumor effects[16], CCL20 produced by tumor cells[17] or immune cells [18] can also create an immunosuppressive tumor microenvironment to support tumor progression. Accordingly, preclinical and clinical studies have suggested that chemokines that mediate interactions within the tumor microenvironment are attractive therapeutic targets to explore to improve response to cancer treatments[19–21]. In this review, how CCL20–CCR6-mediated crosstalk between tumor cells and their microenvironment to enhance tumor progression and treatment outcome has been discussed.

## CCL20 and tumor microenvironment

Tumor development and progression result from the dynamic and complex interaction between tumor cells and their microenvironment which comprises immune cells and non-cellular components[22–25]. Hence, understanding such interactions is important in designing effective strategies that might disrupt these interactions. In the tumor microenvironment, CCL20 enhances the recruitment of immunosuppressive cells including regulatory T cells (Tregs), tumor-associated macrophages (TAMs), tumor-associated neutrophils (TANs), and myeloid-derived suppressor cells (MDSCs) to enhance tumor progression [3, 11, 26]. Besides, CCL20 interacts with non-immune components including endothelial cells [27] and fibroblasts[28] leading to tumor progression as shown in Table 1.

**Table 1:** CCL20-mediated interactions in the tumor microenvironment

Components of tumor microenvironment	Impact on tumor processes	Cancer type	Ref
Tregs	CCL20 promotes the recruitment of Tregs, making tumors resistant to 5-FU Boost Tregs infiltration to enhance cancer stemness and tumor metastasis Enhance infiltration of Tregs leading to tumor progression Enhance eomesodermin-mediated Tregs recruitment and tumor proliferation Drive Tregs infiltration to impair antigen presentation Increases Tregs infiltration to suppress antitumor immunity	Colorectal Colorectal Esophageal Esophageal HCC Lung, Breast Hodgkin's lymphoma cutaneous squamous	[34] [35] [40] [43] [47] [36, 48–50]
Macrophages	Promote macrophage infiltration and polarization to M2, promoting tumor metastasis Promote macrophage-mediated tumor metastasis Facilitate macrophage recruitment and polarization to M2 phenotype to promote proliferation and invasion Enhance M2 macrophage-mediated angiogenesis and immunosuppression Promote macrophage-mediated antitumor suppression Promote M2 macrophage polarization to enhance tumor proliferation	Colorectal, Pancreatic HCC, Breast, Prostate, Renal, ovarian Prostate Lung, Colon, melanoma Cervical, HCC, Prostate Glioma	[53, 62, 63, 65, 66] [71, 72] [64] [55] [67, 68, 73] [56]
MDSCs	Induce PMN-MDSCs to promote breast cancer stemness and drug resistance to docetaxel via CXCL2/CXCR2 Enhance PMN-MDSCs accumulation to promote tumor proliferation and resistance to oxaliplatin Enhance MDSCs accumulation to impair the sensitivity of anti-PD-1	Breast HCC NSCLC	[17] [76] [26]
Neutrophils	Impair antitumor immunity by inducing PD-L1 expression on neutrophils Induce IL17 expression to promote neutrophil infiltration and angiogenesis leading to antitumor suppression of CD8 + T cells and tumor growth	Breast Gastric	[11] [83]
Endothelial Cells	Promote angiogenesis and tumor metastasis Promote endothelial migration, tube formation, and angiogenesis Impair the sensitivity of tumors to crizotinib through VEGFA/IL6-mediated angiogenesis Promote tumor vascularization and growth through HIF-1α upregulation	HCC Melanoma NSCLC Glioblastoma	[84, 85] [86] [27] [87]
Fibroblast	CCL20 derived from stromal fibroblast promotes cancer progression through Th17	Cervical	[28]

## CCL20-mediated interaction between tumor cells and Regulatory T cells (Tregs)

Regulatory T cells are a distinctive lineage of CD4 + T cells that suppresses the immune system by restraining immune responses against self-antigens in autoimmunity and excessive immune-mediated inflammation during infection[29]. In the tumor microenvironment, Tregs are one of the major immunosuppressive cells known to suppress antitumor immunity[30–32]. Evidence shows that the CCL20/CCR6 axis can promote therapeutic resistance, metastasis, and immunosuppression by enhancing the recruitment of Tregs[33].

In colorectal cancer, high expression of CCL20 in cancer cells promotes the recruitment of Tregs, making tumors resistant to 5-FU treatment [34]. In tumor-bearing mice, injection of anti-CCL20 suppressed Treg-mediated tumor growth and sensitized tumors to 5-FU treatment [34].

Ouyang et al. have shown that CCL20 expression induced by RANKL/RANK in colorectal cancer cells can engage the CCR6 receptor to enhance tumor stemness and metastasis by boosting the infiltration of Tregs [35]. Li et al. recently showed that Treg infiltration induced by sideroflexin-mediated CCL20 expression drives immunosuppression and lung adenocarcinoma progression[36].

DNA methylation is a key player in tumor microenvironment interactions [37–39]. In esophageal cancer, Nan et al. found that CCL20 induced by hypomethylation can promote the infiltration of CCR6 + Th17 and Tregs leading to tumor progression[40]. Eomesodermin is a T-box transcription factor that drives lymphocyte differentiation and function[41, 42]. In esophageal cancer, the CCL20–CCR6 axis potentiated eomesodermin-mediated Tregs recruitment and tumor proliferation [43].

Hypoxia can alter the expression of chemokines to shift the tumor microenvironment toward immunosuppressive[44–46]. Suthen et al. showed that hypoxic hepatocellular carcinoma (HCC) cells can upregulate CCL20 expression. Additionally, CCL20 can drive Tregs infiltration to impair antigen presentation of HLA-DR on type 2 conventional dendritic cells, enhancing tumor progression [47]. In inflammatory breast cancer, EGFR regulates the expression of key chemokines including CCL20 to promote tumor progression by increasing the infiltration of Tregs while decreasing CD8 + T cells [48]. A study by Xiao et al. has noted that upregulation of CCL20 in EBV-infected Hodgkin's lymphoma tumor microenvironment promotes the infiltration of Tregs, which in turn suppresses immune responses against EBV-infected tumors[49]. Yuan et al. investigated how miR-22 contributed to Treg-mediated immunosuppression and tumor progression of cutaneous squamous carcinoma [50]. The authors established that miR-22 activated key chemokines in cancer cells including CCL20 to enhance the infiltration of Tregs leading to antitumor suppression of CD8 + T cells[50]

## CCL20-mediated interaction between tumor cells and macrophages

Tumor-associated macrophages are one of the main tumor-infiltrating immune cells known to orchestrate cancer-related processes including tumor proliferation, angiogenesis, therapeutic resistance, metastasis, and immunosuppression[24, 51, 52]. Accumulating studies have shown that CCL20-mediated interaction between tumor cells and macrophages influences several tumor processes including metastasis [53], immunosuppression[54], angiogenesis[55], and proliferation[56].

The microbiome can impact cancer in various ways, one is by promoting an immunosuppressive tumor microenvironment[57, 58]. For example, F. nucleatum enriched in colorectal cancer cells (CRC) can upregulate CCL20. Furthermore, CCL20 activation promoted macrophage infiltration and polarization to M2 phenotype which in turn enhanced CRC metastasis [53]. Emerging evidence has shown that metabolites from microbiota can shape the tumor microenvironment to enhance tumor progression[59–61]. Notably, short-chain fatty acids derived from the gut microbiota promoted the migration of prostate cancer cells through TLR3-mediated autophagy[62]. In the same study, autophagy-induced CCL20 expression in tumor cells further potentiated the invasiveness of prostate cancer cells by promoting the infiltration and polarization of M2 macrophages[62].

CCL20 activated by vitamin D receptor in pancreatic cancer cells enhanced tumor progression through CCL20-mediated macrophage recruitment and polarization toward M2 phenotype[63]. Similarly, circSMARCC1-mediated CCL20 expression facilitated macrophage recruitment, thus promoting tumor progression [64]. Gao et al. noted that autophagy inhibition can promote CCL20/CCR6-mediated infiltration of macrophages and HCC metastasis via Ilβ signaling [65].

Studies have shown that cancer cells undergoing epithelial‐mesenchymal transition (EMT) can contribute to immunosuppressive tumor microenvironment. A recent explored the effect and molecular mechanisms of cathepsin D (Cat D) in the tumor microenvironment and showed that Cat D-mediated expression of CCL20 in cancer cells can promote polarization of M2 macrophages enhancing epithelial mesenchymal transition (EMT) and tumor metastasis[66]. In cervical cancer, CCL20 promoted an immunosuppressive tumor microenvironment by enhancing M2 macrophage infiltration while inhibiting CD8 + T cell infiltration [67]. According to Ye et al. hypoxic HCC cells undergoing EMT can increase CCL20 expression. Additionally, indoleamine 2,3-dioxygenase(IDO) induced in a CCL20-dependent manner by coculturing hypoxic HCC cells with monocyte-derived macrophages suppressed T cells and promoted HCC progression [68]. Therapeutic agents can shape tumor cells and their microenvironment to promote tumor progression. Hence, studies exploring the mechanisms of therapeutic-driven tumor progression present new opportunities for effective treatments [69, 70]. For example, Liu et al. explored the effects of chemotherapy on the immunosuppressive tumor microenvironment and found that cisplatin treatment can induce classically activated macrophages (CAMs) to express CCL20. CAMs-induced CCL20 engages with its receptors CCR6 leading to EMT and ovarian cancer metastasis [71]. In renal cell carcinoma tumor microenvironment, macrophage-mediated CCL20 via AKT activation promoted the migration of cancer cells through EMT induction[72].

In prostate cancer expression, CCL20 in macrophages and monocytes was found to suppress antitumor immunity of T cells leading to bone marrow metastasis [73]. In syngeneic tumor-bearing mice, CCL20/CCR6 distortion rejuvenated T cell activity and prolonged the survival of tumor-bearing mice [73].

Using lung cancer, melanoma, and colon cancer mouse models, ADAR1 secretion in macrophages contributed to the establishment of immunosuppressive tumor microenvironment via CCL20-mediated angiogenesis and antitumor suppression. The same study noted that loss of ADAR1 led to a corresponding decrease in CCL20 expression, inhibited angiogenesis, and boosted antitumor immunity of CD8 + T cells[55]. In glioma, CCL20 upregulated by ZNF395 enhanced M2 macrophage polarization which in turn promoted the proliferation of glioma cells [56].

## CCL20-mediated interaction between tumor cells and myeloid-derived suppressor cells (MDSCs), and neutrophils

Myeloid-derived suppressor cells consist of monocytic and polymorphonuclear immature myeloid cells whose expansion during cancer suppresses antitumor immunity leading to tumor progression[74, 75]. It has been shown that high expression of CCL20 in breast cancer cells can potentiate the differentiation and expansion of granulocyte-monocyte progenitors into polymorphonuclear myeloid-derived suppressor cells (PMN-MDSCs) via CCR6. Furthermore, CCL20-induced PMN-MDSCs increased breast cancer stemness and drug resistance to docetaxel via CXCL2/CXCR2 [17]**.** Liu et al. explored the underlying mechanism of oxaliplatin resistance and immune evasion of HCC cells and found that DDR2/STAT3-mediated CCL20 expression can drive PMN-MDSCs accumulation and promote HCC proliferation and resistance to oxaliplatin [76]. In non-small cell lung cancer (NSCLC), CCL20 upregulation by METTL3 promoted infiltration of several immunosuppressive cells including MDSCs to impair the sensitivity of tumors to anti-PD-1 immunotherapy [26]. In glioma, Chang et al. identified MDSCs as one of the immune cells induced through the CCL20/CCL2 axis to promote an immunosuppressive tumor microenvironment [77].

Tumor-derived factors including chemokines are crucial in mediating the interaction between tumor cells and neutrophils [78–82]. Using deep immunophenotyping at the single-cell level, Nagaoka et al. showed that CCL20-mediated IL17 in gastric cancer cells can promote the infiltration of neutrophils and angiogenesis leading to antitumor suppression and tumor growth [83]. Kwantwi et al. have shown that breast cancer-derived CCL20 can impair antitumor immunity by inducing PD-L1 expression on neutrophils[11].

## CCL20-mediated interaction between tumor cells and endothelial cells and fibroblast

As a major component of the tumor microenvironment, endothelial cells promote blood vessel formation to support tumor growth and metastasis to distant organs[88]. Emerging evidence has shown that CCL20 and its receptor CCR6 are important players in endothelial cell-mediated tumor progression. In HCC, CCR6 derived from exosomes enhanced metastasis and angiogenesis. In the same study, CCL20 upregulated by HOXD3 promoted tumor metastasis and angiogenesis [85]. In NSCLC, CCL20 activated through STAT3 signaling impaired the sensitivity of tumor-bearing mice to crizotinib via VEGFA/IL6-mediated angiogenesis [27]. High expression of CCL20 in tumor cells is associated with endothelial migration, tube formation, and angiogenesis [86]. Furthermore, tumor vascularization and growth were significantly decreased in CCR6-deficient mice [86]. In immunocompetent HCC tumor-bearing mice, CCL20 neutralization not only suppressed tumor metastasis but also inhibited angiogenesis[84]. Benkheil et al. found that CCL20 induced by hepatitis c virus (HCV) in HCC cells can interact with CCR6 expressed in endothelial cells to promote hepatic angiogenesis [89]. In colorectal cancer, VEGF expression induced by CCR6 through AKT/NF-κB promoted angiogenesis [90]. According to Jin et al. CCL20 secreted by hypoxic glioblastoma astrocytes can contribute significantly to tumor vascularization and growth through HIF-1α upregulation[87].

Fibroblasts are a significant component of the tumor microenvironment that plays a crucial role in tumor progression[91, 92]. Increasing evidence has shown that chemokines including CCL20 are important orchestrators of fibroblast-mediated cancer progression. In cervical cancer, CCL20 derived from stromal fibroblast promoted the progression of cervical cancer through CCL20-mediated recruitment of Th17[28].

## CCL20 in combination therapies

CCL20 has become an important target for cancer therapies. Although the development of drugs modulating the CCL20/CCR6 axis is expected but currently not available[93], blocking the CCL20–CCR6 axis in combination with standard cancer therapies has shown promising results. Nanoparticles that utilize tumor microenvironment characteristics have shown promise in overcoming multidrug resistance, hence changing the perspective of cancer treatment[94, 95]. As chemokines are best noted for recruiting immune cells, CCL20 has been used in combination with nanoparticles. For example, Da Silva et al. designed a biodegradable nanoparticle and used it as a delivery vehicle for doxorubicin, two immune adjuncts, and CCL20 for treating resistant TC-1 lung carcinoma and MC-38 colon adenocarcinoma. The study found that this multidrug nanoparticle induced high circulating and tumor infiltration of cancer-specific T cells leading to improved survival of tumor-bearing mice than each treatment alone[96].

Identification of factors driving the expression of immune checkpoint molecules and their associated antitumor suppression has resulted in therapeutic approaches mitigating the expression of this immunosuppressive checkpoint leading to improved efficacy of immune checkpoint blockade. In TME, CCL20 regulates not only immune tracking but also PD-L1-mediated immunosuppression and hence a key therapeutic potential for checkpoint blockade [26, 97]. In HCC cells that are resistant to oxaliplatin, a combination of anti-CCL20 and anti-PD-L1 reduced the infiltration of MDSCs while increasing the accumulation of CD8 + T cells, thereby inhibiting HCC progression [76]. Rutihinda et al. investigated factors contributing to radiotherapy resistance and how to improve treatment outcomes [98]. The study found that treating head and neck squamous cell carcinoma (HNSCC) with radiation promoted CCL20 expression in HNSCC which in turn facilitated Tregs accumulating leading radiation resistance and tumor growth. On the other hand, CCL20–CCR6 inhibition suppressed tumor growth by inhibiting infiltration of Tregs. This effect was further enhanced using CCL20 inhibition and radiotherapy than each treatment alone[98].

## Conclusion

This review has shown the significant role of the CCL20–CCR6 axis in tumor microenvironment interactions and their implication in cancer progression. This increasing knowledge will open the door to developing effective anticancer combination therapies that can reverse CCL20-mediated chemotaxis of immunosuppressive cells including neutrophils, macrophages, Tregs, and MDSCs while enhancing immune-mediated destruction of cancer. However, the molecular and regulatory mechanisms of CCL20 in the tumor microenvironment are complex, and more rigorous research is needed.
